# Risky Alcohol Use and Comorbidity in a Swedish Adolescent Emergency Psychiatric Inpatient Population

**DOI:** 10.1155/2021/5514144

**Published:** 2021-10-19

**Authors:** Sophia Eberhard, Olof Rask, Peter Höglund, Maria Råstam, Björn Axel Johansson

**Affiliations:** ^1^Department of Clinical Sciences Lund, Child and Adolescent Psychiatry, Lund University, SE-221 85 Lund, Sweden; ^2^Department of Clinical Sciences Lund, Clinical Chemistry and Pharmacology, Lund University, SE-221 85 Lund, Sweden; ^3^Region Skåne, Psychiatry and Habilitation, Child and Adolescent Psychiatry, Regional Inpatient Care, Emergency Unit, SE-205 02 Malmö, Sweden; ^4^Gillberg Neuropsychiatry Centre, Institute of Neuroscience and Physiology, University of Gothenburg, SE-413 45 Gothenburg, Sweden

## Abstract

**Introduction:**

Underage drinking is associated with poor mental health. Early detection for risky alcohol use is recommended, although less implemented in adolescent care. The objective of this study was to investigate the prevalence of risky alcohol use and psychiatric comorbidity.

**Methods:**

Over a nine-month period, 145 eligible adolescents admitted to the child and adolescent emergency unit in Malmö, Sweden, were offered computerized screening with Alcohol Use Disorder Identification Test-Consumption (AUDIT-C) and Drug Use Disorders Identification Test (DUDIT).

**Results:**

Ninety-six patients (73 girls and 23 boys) agreed to participate. The most common diagnoses were affective disorder (41%) and anxiety disorder (27%). Risky alcohol use was found among 33% of the girls and 22% of the boys (*p*=0.45) and did not differ between diagnostic categories. There was a positive correlation between AUDIT-C and DUDIT scores (*p*=0.019). Among adolescents with risky alcohol use, 33% of the girls and 60% of the boys also had a risky drug use. Conversely, 47% of the girls and 60% of the boys with risky drug use also had a risky alcohol use.

**Conclusion:**

In view of the poor prognosis of risky alcohol use in adolescents, alcohol and drug habits should be assessed when adolescents seek psychiatric emergency care.

## 1. Introduction

Underage drinking is a serious public health problem associated with poor mental health in general and specifically with depression, anxiety, and antisocial and aggressive behaviour [1, 2]. A more severe condition, alcohol use disorder (AUD), is associated with alcohol-related injury, illicit drug use, and mortality [3, 4]. Adolescents who start to drink before the age of 15 are significantly more likely to develop alcohol dependence in adulthood than those who delay their debut until 18 or older [5].

In Sweden, alcoholic beverages can be legally purchased from the age of 20, and in bars and restaurants, alcoholic beverages may be ordered from the age of 18. Nevertheless, in a national survey of adolescents aged 15-16 years, 40% reported use of alcohol during the past year [6], and Strandberg and coworkers [7] found that, among adolescents and young adults aged 15–27 years visiting youth health clinics in Stockholm, Sweden, 62% reported a “risky alcohol use.” In a longitudinal study, Danielsson et al. found that one episode or more of heavy drinking, i.e., binge drinking, predicted continued irregular heavy drinking two years later in boys, and the same trend was shown for girls [8]. Binge drinking is an important aspect of risky alcohol use, strongly linked to fights and withdrawal symptoms. There is no clear lower threshold for when alcohol consumption is not associated with a risk of disease [9], but a scoring instrument is often used to describe drinking patterns and grading risky behaviour [2, 4, 10]. Risky alcohol use in general, and binge drinking in particular, is a risk factor for suicide and suicide attempts and for developing a substance use disorder (SUD) [11].

A psychiatric disorder more than doubles the lifetime risk for developing AUD [12], and comorbid AUD in psychiatric patients worsens outcome and prolongs psychiatric disorder duration [13]. Early identification and treatment of AUD are associated with better psychiatric, psychosocial, and drug-related outcome [14, 15]. This highlights the importance of early detection, as a way of preventing risky alcohol use. In international guidelines, screening, brief intervention, and referral to treatment are recommended, although less implemented in adolescent healthcare [16, 17]. Davis et al. [2] suggested that a consultation to an emergency unit offering information about alcohol could represent an important “teachable moment” for young patients and makes it a good setting for screening, short interventions, and referral to treatment. Computerized questionnaires may potentially be an efficient method that could easily be implemented in child and adolescent psychiatric care [18].

In this context, we hypothesized that alcohol use could be common among adolescents with psychiatric emergencies, with a possibly heterogenous distribution among different diagnostic categories. The objectives of the present study were to investigate the prevalence of risky alcohol use in a Swedish child and adolescent emergency psychiatric inpatient population and to study the association between risky alcohol use and psychiatric comorbidity, including risky drug use.

## 2. Materials and Methods

### 2.1. Participants

Over a nine-month period (June 2017–February 2018), adolescents consecutively admitted to the regional child and adolescent emergency unit in Malmö, Sweden, who did not fulfil any exclusion criteria, were offered screening of their alcohol and drug habits. The unit's catchment area, Skåne in southern Sweden, has a population of over one million people, of which 200,000 are children and 80,000 are adolescents. Every year, approximately 400 unique patients, accompanied by a parent or caregiver, are admitted for emergency inpatient psychiatric care.

**Figure 1:**
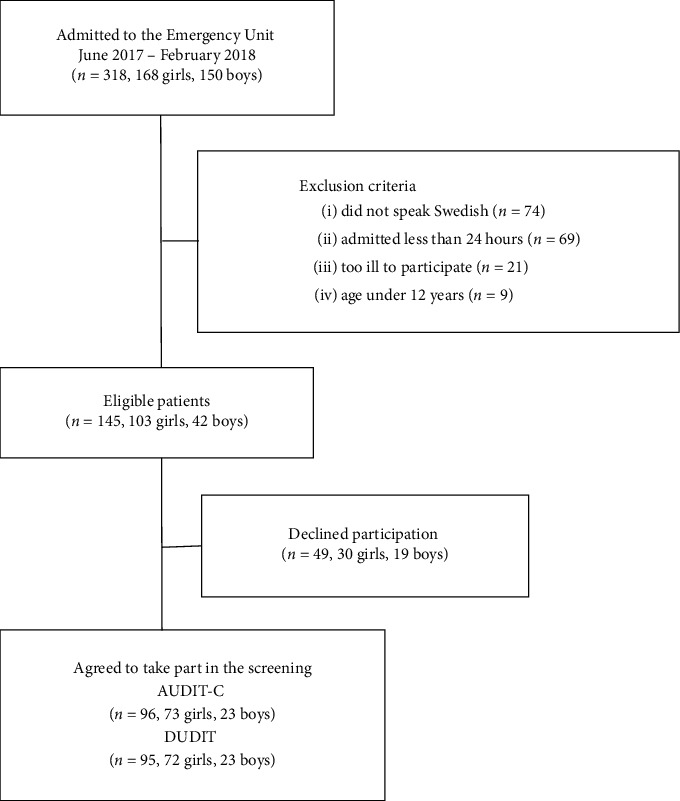
Patient flow diagram.

All patients admitted to the emergency unit that did not meet any exclusion criteria were eligible for participation in the study (Figure 1). Decisions regarding eligibility, during the daily rounds and based on the inclusion and exclusion criteria, were made by the senior consultants at the unit. Patients were excluded if not able to understand Swedish (questionnaires not available in other languages), discharged less than 24 hours from arrival (practical reasons, e.g., admitted and discharged the same night), with severe psychiatric illness, e.g., psychotic disorders (thus considered not being able to take a well-founded position on participation or not being able to provide accurate answers), or if younger than 12 years (preadolescents). Recruitment to the study was done during the patients' treatment period by dedicated staff to whom all patients and their legal guardians gave their informed consent, which was electronically registered in the screening tool “The Blue App” [19]. Participating adolescents completed two computerized self-rating questionnaires: Alcohol Use Disorder Identification Test-Consumption (AUDIT-C) [20, 21] and Drug Use Disorders Identification Test (DUDIT) [22]. Compensation for participation was not given.

One hundred forty-five patients qualified for inclusion. Of these, 96 patients (66%) agreed to participate and completed AUDIT-C and 95 completed DUDIT. Data were collected regarding main psychiatric diagnoses, age, gender, length of the hospital stay, and any coercive treatment. Main diagnoses were classified and categorized into diagnostic groups according to The International Statistical Classification of Diseases and Related Health Problems (ICD-10) by senior consultants in child and adolescent psychiatry after evaluation and discussion in a multiprofessional team [23]. Eleven patients were admitted more than once during the study period. For these, screening results from their first admission were used.

### 2.2. Instruments

Alcohol Use Disorders Identification Test (AUDIT) is a screening instrument for hazardous and harmful alcohol consumption and possible dependence [24]. AUDIT consists of ten questions regarding hazardous alcohol consumption, frequency of intoxication, drinking patterns, and adverse consequences, respectively, and has been extensively used in adult target groups. The maximum score for each item is 4, giving a total maximum score of 40. AUDIT has been translated into Swedish and validated with satisfactory internal and test-retest reliability [21].

There is growing support for AUDIT-C in identifying risky alcohol use among adolescents. This is a three-item derivate of AUDIT with emphasis on consumption rather than the negative consequences of drinking [2, 25]. In the recent literature, the term “hazardous alcohol use” is often replaced by “risky alcohol use.” AUDIT-C was used to measure alcohol consumption and consumption patterns, i.e., frequency, quantity, and binge drinking, defined as >5 drinks on any occasion at least once a month. Each question in the AUDIT-C is scored from 0 to 4, giving a maximum score of 12 points. As suggested in some studies on adolescents, we used the cutoff for risky alcohol use of ≥3 in total score for both girls and boys [2, 4].

Drug Use Disorders Identification Test (DUDIT) is a screening instrument for detecting drug-related problems, subsequently translated to Swedish and validated [22, 26]. The questionnaire comprises 11 questions. The maximum score for each item is 4, giving a total maximum score of 44. In a study on the general Swedish population, the preliminary recommendation is a cutoff score of ≥2 for women and ≥6 for men to identify harmful and hazardous drug use [22]. As with risky alcohol use, these cutoff scores are used to define risky drug use in the present study.

### 2.3. Data Collection

The screening questionnaires were administered electronically on either the patients' smartphone or on the ward tablets, using a web-based mobile phone application for electronic data collection, “The Blue App,” developed by our research group as previously described [19]. Two dedicated staff members were recruited to assist in the screening process, mainly providing information about the study and helping to start up the software.

### 2.4. Statistical Analysis

The Mann–Whitney *U* test was applied for continuous data. Differences in frequencies were analysed with the chi-square test and Fisher's exact test. The level of significance was set to *p* < 0.05. Reliability data were assessed for AUDIT-C, Cronbach's alpha >0.7 considered acceptable. Statistical analyses were carried out using SPSS ver. 24.

### 2.5. Ethical Approval

The study complies with the guidelines of the Committee of Ethics in Sweden. Prior to data collection, the study protocol was reviewed by the Lund Regional Ethics Board. The board approved the study as a clinical quality project (No. 423/2013) and did not see any potential risks or barriers to the project from an ethical point of view.

## 3. Results

### 3.1. Demographics

The study sample (*n* = 96) consisted of 73 girls (76%) and 23 boys (24%). The median age was 15.8 (14.9–16.8) years for girls and 16.4 (15.2–17.2) years for boys. No significant differences were found between boys and girls in terms of age distribution, diagnoses, or duration of the inpatient stay.

The study sample did not significantly differ from the 49 decliners in terms of distribution of diagnoses, except for affective disorder (49% in our sample and 31% of the decliners; *p*=0.035) and autism (11% in our sample and 0% of the decliners; *p*=0.025). No significant differences were found regarding age, gender, or length of the hospital stay. Coercive treatment was zero in the decliners, and 10% among those included (*p*=0.027).

### 3.2. Risky Alcohol Use and Binge Drinking

Table 1 shows the distribution of AUDIT-C scores in the sample. Reliability as measured by Cronbach's alpha (0.86) was satisfactory. The average (SD) score was 1.8 (2.6) for girls and 1.3 (2.0) for boys. Twenty-four of 73 girls (33%) had AUDIT-C scores indicating risky alcohol use, compared with five of 23 boys (22%) (*p*=0.45). Adolescents with risky alcohol use were older compared to those with scores below cutoff; for girls, average ages were 16.0 years versus 15.1 years (*p*=0.031), and for boys, 16.6 years versus 15.3 years (*p*=0.31). Forty-two percent of the girls and 39% of the boys reported use of alcohol at some time during the previous 12 months.

Sixty-seven of the 96 patients scored 0–2 points, i.e., 70% of the patients scored below cutoff for risky alcohol use. Fifty-eight percent of all patients (*n* = 56) presented AUDIT-C scores of zero, i.e., reported no use of alcohol. The proportion of abstinence in boys (61%, *n* = 14) was similar to that in girls (58%, *n* = 42). Seven patients (7 girls, 0 boys) fulfilled the definition of binge drinking.

### 3.3. Risky Drug Use

The results of the DUDIT screening are presented in Table 2. Screening was completed by 72 of the 73 girls and by all 23 boys. The average (SD) score was 1.8 (3.9) for girls and 2.2 (4.7) for boys. No significant difference in distribution of DUDIT scores was found between boys and girls (*p*=0.14). Seventeen of the 72 girls (24%) and five of the 23 boys (22%) had DUDIT scores indicating risky drug use. Seventy-six percent of the patients presented DUDIT scores of zero, indicating no drug use.

### 3.4. Comorbidity

The main diagnoses are listed in Table 3. The most common diagnoses were affective disorder (41%, *n* = 39) and anxiety disorder (27%, *n* = 26) (Table 3). Risky alcohol use was found in all diagnostic categories, except for eating disorders (anorexia nervosa). The proportion of risky alcohol use did not differ significantly between the categories (*p*=0.52).

**Figure 2:**
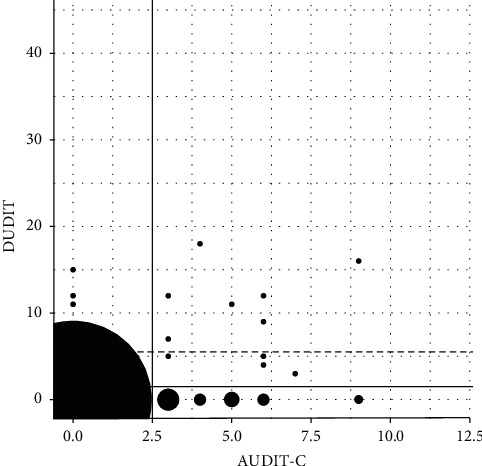
AUDIT-C and DUDIT scores. Black areas represent number of observations. Cutoffs for risky alcohol use (AUDIT-C ≥ 3, vertical line) (*n* = 96) and risky drug use (DUDIT ≥6 (boys) and DUDIT ≥2 (girls), horizontal lines) (*n* = 95) are shown.

A positive correlation between AUDIT-C and DUDIT scores was found (rho 0.24; *p*=0.019) (shown in Figure 2). Eight of the 24 girls with risky alcohol use (33%) also had a risky drug use, and three of the five boys with risky alcohol use (60%) also had a concurrent risky drug use. Conversely, of the 22 patients with scores indicating risky drug use, eight girls (47%) and three boys (60%) also scored above cutoff in the AUDIT-C, indicating a concurrent risky alcohol use (OR = 3.0, CI = 1.0–9.2, *p*=0.034).

## 4. Discussion

This study systematically and prospectively evaluated adolescents admitted to psychiatric emergency care. Risky alcohol use was found in one in three girls and one in five boys. Comorbid risky drug use was common. Risky drug use was found in almost a quarter of the study population, also with a high degree of concurrent risky alcohol use.

Few studies have examined the impact of risky alcohol on child and adolescent emergency services. We have not found any strictly comparable studies with emergency admission psychiatric adolescents evaluated with AUDIT-C and DUDIT. In some studies, the patients have been older [25], treated in open care [4], or belonged to the general population, while other studies have involved children seeking somatic emergency care [25]. In a large US study from 2006, prevalence rates of alcohol use disorders among adolescents who used mental health services were 6% for abuse and 4% for dependence, respectively [27].

### 4.1. Risky Alcohol Use

AUDIT-C is validated for young people and competitively compared with other screening forms, e.g., Fast Alcohol Screening Test (FAST) and CRAFFT (acronym for Car, Relax, Alone, Forget, Friends, Trouble) [28, 29]. In studies using AUDIT-C in adolescents, the age of the study population has varied, as well as the clinical context and cutoffs; both 4 and 5 have been used to identify adolescents and young adults with alcohol problems [4]. Kelly et al. [25] examined 181 young adults aged 18–20 who had visited a somatic emergency room. Their average AUDIT-C score was 5.0 (SD: 2.7). Liskola et al. [4] examined 218 depressed 16-year-olds treated in psychiatric open care. Their average AUDIT-C score was 4.2 (SD: 3.0). For early identification of risky alcohol use, a cutoff score of ≥3 was recommended. In clinical practice, especially in early adolescence, we however recommend that any score above zero should be considered possibly harmful and interventions should be considered.

Davis et al. [2] mapped drinking habits using three different screening forms in nonpsychiatric patients aged 14–20 visiting an emergency department in the US and found risky alcohol use in 24% of the patients. Knight et al. [29] screened nonpsychiatric patients aged 14–18 in hospital-based adolescent primary care, finding risky alcohol use in 28% of the patients. In the present study of strictly adolescents, 30% reported a risky alcohol use, indicating that alcohol consumption is also a serious problem among adolescents with psychiatric emergencies.

Alcohol consumption and drinking patterns among students in school years 9 and 11 in Sweden are annually screened by the Swedish Council for Information on Alcohol and Other Drugs (CAN) [6]. In the 2016 survey, 40% of adolescents aged 15–16 years reported use of alcohol during the past year. In our study, we assessed a clinical emergency sample and found that 42% of the girls and 39% of the boys reported use of alcohol at some time during the previous 12 months. In this more general aspect, our results seem to be representative of the Swedish adolescent population.

Responses to question three (“How often do you have six or more drinks on one occasion?”) from AUDIT-C were analysed separately, as binge drinking has been proposed to be the most dangerous aspect of risky alcohol use and a strong risk factor for a variety of drink-related complications [9, 30]. Seven percent of the patients, all girls, recorded scores indicating binge drinking. The result indicates that binge drinking is a major problem, echoing some previous studies [8]. The gender difference, albeit the small sample size, is in line with recent findings by Anderberg and Dahlberg, who demonstrated that girls treated in outpatient clinics for SUD have more substance use compared to boys [31]. In another Swedish study, it was shown that the prevalence of risky drinking in twins, with and without an autism proxy diagnosis, was higher among girls at the age of 15 years [32]. In the US, studies have found an increased risk for females in early-mid adolescence of becoming underage drinkers [33], a possible reason for this might be changes in gender expectations. The results from the present study further highlight the clinical relevance of screening for risky alcohol use.

We argue that the cutoff for binge drinking recommended in AUDIT-C, >5 drinks on any occasion at least once a month, is too high for adolescents who, by law, should not be drinking alcoholic beverages [34]. A lower cutoff of 4 or more drinks has been proposed [6, 16]. In the CAN survey, 8% of 15-16-year-olds reported drinking four or more drinks on a single occasion. As the CAN survey uses a different definition for binge drinking than AUDIT-C and does not map other aspects of risky alcohol use, comparable data are missing. Our findings indicate that binge drinking could be more severe among psychiatric inpatients than in the general Swedish adolescent population.

### 4.2. Comorbidity

Regardless of main diagnosis, our patients were at risk of developing SUD [3, 4]. The high prevalence of risky alcohol use found indicates the importance of screening for SUD in a child and adolescent psychiatric emergency population [35]. Anxiety and affective disorders represent the majority of patients admitted to the unit. Our patients with neuropsychiatric disorders, i.e., autism and attention deficit hyperactivity disorder (ADHD), also had a high prevalence of risky alcohol use. This is in line with findings in other studies, which reported that ADHD increases the risk for AUD, and treatment of ADHD has been proposed to diminish the risk of developing an SUD comorbidity [36, 37]. ADHD with comorbid autistic traits seems to be at particularly high risk [38]. We found no risky alcohol use among our patients with eating disorders. The reason for this is likely that our patients were treated for anorexia nervosa which, among adolescents, unlike bulimia, is associated with low alcohol consumption [39, 40].

Risky drug use was found in almost a quarter of the study population, also with a high degree of concurrent risky alcohol use. A positive correlation between risky alcohol use and risky drug use (*p*=0.019) was found. One explanation could be that adolescents who drink alcohol possibly socialize in the same circles as people using other drugs [41]. Considering the high prevalence of risky alcohol and drug use found in our study, a higher proportion of SUD might have been expected as main diagnoses. One of the reasons we did not find this could be that patients in pediatric psychiatry usually do not seek emergency care for SUD as their main complaint, but rather because of other problems such as depression and anxiety. SUD might be found among the bidiagnoses that should be addressed. This emphasizes that when adolescents are admitted for psychiatric emergency care, no matter the cause, an evaluation of both risky alcohol and drug use is important.

### 4.3. Limitations

The study design did not allow us to compare the results on AUDIT-C and DUDIT with the patients' toxicological screening tests, nor record the type of drugs that were used. The exclusion criteria may have affected the results; subjects excluded because of language difficulties were to a vast majority unaccompanied minors from middle east countries where alcohol is used to a limited extent, and subjects excluded due to admittance for less than a day could be assumed to be less affected by serious psychiatric illness, e.g., depression, which in turn is a risk factor for risky alcohol and drug use. These subgroups thus need to be further studied. The screening instruments AUDIT-C and DUDIT are validated and have shown satisfactory psychometric properties, but it is not clear if other screening instruments may perform better in terms of screening adolescent populations.

### 4.4. Clinical Implications and Future Research

This study provides information about risky alcohol use and its comorbidity that could serve as epidemiological foundation for social policies, interventions, and future research. The study findings clearly indicate that it is both possible and essential to identify substance-related problems among adolescents seeking psychiatric emergency care for other reasons [42]. The study used a recently developed method for computerized data collection [19], demonstrating a promising feasibility for use in clinical practice. In view of the correlation between AUDIT-C and DUDIT scores, it is important to take risky drug use into consideration when admitting adolescents for psychiatric emergency care and to plan for relevant support in the emergency setting and subsequent interventions. The findings extend earlier research and should be useful for a better clinical understanding and care for this vulnerable group. A key approach in future research would be a longitudinal follow-up and study of treatment interventions.

## 5. Conclusions

Adolescents admitted to psychiatric emergency care often present risky alcohol and drug use in addition to their main diagnosis. In view of the poor prognosis of risky alcohol use in adolescents, especially with psychiatric comorbidity, alcohol and drug habits should be assessed as part of good clinical practice when treating adolescents. Emergency units seem to be suitable for computerized screening.

## Figures and Tables

**Table 1:** AUDIT-C scores from the screening.

AUDIT-C scores	0	1	2	3	4	5	6	7	8	9	10–12	Risky alcohol use
Girls, *n* (%)	42 (58)	4 (5)	3 (4)	**8 (11)**	**3 (4)**	**4 (5)**	**5 (7)**	**1 (1)**	**0 (0)**	**3 (4)**	**0 (0)**	**24 (33)**
Boys, *n* (%)	14 (61)	3 (13)	1 (4)	**1 (4)**	**1 (4)**	**1 (4)**	**2 (9)**	**0 (0)**	**0 (0)**	**0 (0)**	**0 (0)**	**5 (22)**

The scores in bold indicate risky alcohol use (≥3). Total sample *n* = 96, 73 girls and 23 boys.

**Table 2:** DUDIT scores from the screening.

DUDIT scores	0	1	2	3	4	5	6	7	8	9	10–18	19–44	Risky drug use
Girls, *n* (%)	55 (76)	0 (0)	**1 (1)**	**1 (1)**	**4 (6)**	**2 (3)**	**0 (0)**	**2 (3)**	**1 (1)**	**0 (0)**	**6 (8)**	**0 (0)**	**17 (24)**
Boys, *n* (%)	18 (78)	0 (0)	0 (0)	0 (0)	0 (0)	0 (0)	**2 (9)**	**0 (0)**	**0 (0)**	**1 (4)**	**2 (9)**	**0 (0)**	**5 (22)**

The scores in bold indicate risky drug use (≥2 for girls and ≥6 for boys). Total sample *n* = 95, 72 girls and 23 boys.

**Table 3:** Distribution of psychiatric ICD-10 main diagnoses in correlation to risky alcohol use.

ICD-10 diagnostic groups	*n*	Risky alcohol use, *n* (%)
F10–F19, substance use disorders	4	1 (25.0)
F30–F39, affective disorders	39	13 (33.3)
F40–F48, neurotic disorders	26	8 (30.8)
F50–F59, behavioral syndromes	8	0 (0.0)
*F50.0*, *anorexia nervosa*		
F80–F89, developmental disorders	7	2 (28.6)
*F84.0*, *autism*		
F90–F98, behavioral and emotional disorders	9	4 (44.4)
*F90.0*, *ADHD*		
F99, other diagnoses	3	1 (33.3)
Total	96	29 (30.2)

Total sample *n* = 96, 73 girls and 23 boys.

## Data Availability

The full dataset is available from the corresponding author on request.
